# Coffee Roast Level, Timing, and Carbohydrate Source Affect Peak Blood Glucose and Area Under the Curve Values in a Randomized Pilot Clinical Trial

**DOI:** 10.1155/jnme/4174563

**Published:** 2025-10-23

**Authors:** Lina Maria Rayo-Mendez, Craig Kinzer, Jonathan McMahon, Magaritte Nguyen, Gabriel Keith Harris

**Affiliations:** ^1^Department of Food, Bioprocessing, and Nutrition Sciences, North Carolina State University, Raleigh, North Carolina, USA; ^2^Department of Statistics, North Carolina State University, Raleigh, North Carolina, USA

**Keywords:** area under the curve, blood sugar, caffeine, coffee, roasting degree

## Abstract

Roasted coffee's bioactive compounds may affect human glucose metabolism. This pilot clinical trial investigated the impact of coffee roast level, coffee timing, and carbohydrate (CHO) type on blood glucose responses. Healthy participants (15 female and 4 male) completed this six-week, randomized, single-blinded study. Treatments comprised combinations of roast levels (light, medium, or dark), coffee timings (pre-, co-, or post-CHO ingestion), and CHO types: oral glucose tolerance test (OGTT) beverages or a standardized breakfast designated as PreO, PreB, CoO, CoB, PostO, and PostB. Subjects consumed 300 mL of test beverages and provided 10 capillary blood samples over 4 h. Coffees were analyzed for caffeine, 3, 4, and 5 chlorogenic acids (CGA), trigonelline, total phenols, color, °Brix, and total dissolved solids (TDS). Significant (*p* < 0.05) differences in 3CQA, 5CQA, and trigonelline, but not caffeine and 4CQA, were observed across roast levels. Prior to CHO, coffee did not increase blood glucose, but potentiated increases after CHO. PreO and PreB treatments produced the highest peak glucose values (160 mg/dL). This was not observed for co- or posttreatments. In the CoO combination, AUC values were significantly (*p* < 0.05) lower than placebo only for medium roast. In posttreatments, peak glucose levels were higher following OGTT than breakfast, but AUCs did not differ. Light roast coffee yielded the lowest AUC for PreO and the highest peak at 3 h in the CoO combination. Medium roast resulted in the lowest AUC across all time points in CoO, while both medium and dark roasts elevated AUC in PreO. Only dark roast increased AUC in the PreB condition. Three AUC main effects (CHO type, coffee roast, and timing) and two interactions (timing × roast; timing × CHO × roast) were highly significant (*p* < 0.002). These findings may benefit those desiring temporary blood glucose elevations for exercise performance and recovery versus those seeking to moderate glucose.

**Trial Registration:** ClinicalTrials.gov identifier: NCT05119153

## 1. Introduction

Coffee is one of the most widely consumed beverages in the world and has been extensively studied for its potential effects on metabolic health [1–3]. Epidemiological studies suggest an inverse association between habitual coffee consumption and the risk of developing type 2 diabetes mellitus (T2DM) [2, 4, 5]. Paradoxically, some acute studies of caffeine and or coffee intake have reported impairments in glucose regulation, even in healthy individuals [6]. Caffeine, the primary methylxanthine stimulant compound in coffee, has been shown to acutely influence glucose metabolism and insulin sensitivity. Several studies have reported that consuming coffee or caffeine before a carbohydrate (CHO) meal can lead to a higher postprandial blood glucose response [7–14]. Reported causes include catecholamine release, resulting in reduced peripheral glucose uptake and increased hepatic glucose production [15, 16]. One of the principal mechanisms underlying catecholamine release is the antagonistic action of caffeine on adenosine receptors. By inhibiting adenosine receptors, caffeine disrupts insulin-mediated glucose uptake, thereby contributing to elevated postprandial blood glucose levels [3, 7, 15, 17]. These findings underscore the importance of considering caffeine intake in dietary strategies for managing blood glucose. In contrast, others have reported neutral or moderating effects of coffee on glucose regulation, depending on factors such as timing of coffee and carbohydrate intake, carbohydrate type, total caffeine intake, and previous exposures to caffeine [9, 14, 18]. These inconsistencies of reported effects suggest that additional studies are needed to define the interactions of coffee and carbohydrates on blood glucose regulation in humans.

While most coffee-related studies focus on links between caffeine's stimulant properties and its effects on blood glucose, it is important to understand that although caffeine is the most recognized bioactive in coffee, the beverage is a complex mixture of compounds [19] that vary across green roast degree such as light, medium, and dark roasts. The roast degree strongly affects both the color and chemical composition of roasted beans and brewed coffee [20, 21]. Although roasting is desirable for aroma and flavor development in coffee beans due to the accumulation of Maillard reaction products, some potentially bioactive components, such as chlorogenic acids (CGAs) and trigonelline, degrade at higher roasting temperatures while caffeine remains stable [20, 22–25]. The extent to which coffee roast level influences the presence and bioactivity of these compounds and how those chemical changes may affect glucose metabolism remains an open question. To date, no clinical trial has examined the combined effects of coffee roast level, timing of consumption, and carbohydrate type on postprandial blood glucose and area under the curve (AUC) in humans. Therefore, this randomized, single-blind pilot study aimed to evaluate how these three variables interact to influence postprandial glycemic responses in healthy humans following either an oral glucose beverage or a standardized breakfast meal.

## 2. Materials and Methods

### 2.1. Study Subjects

This study was approved by the North Carolina State University Institutional Review Board (IRB protocol 16857) and subsequently conducted on the NC State main campus in Raleigh, NC. This trial was registered at the NIH Clinical Trials Registry (clinicaltrials.gov) under the name, “Effects of Coffee Roasting on Blood Sugar Levels in Healthy Humans.” Healthy male and female subjects between 18 and 65 years of age were candidates for the study. Inclusion criteria included a body mass index (BMI) between 18.6 and 25 kg/m^2^, waist-to-hip ratios of 0.84 for women and ≤ 1 for men, and a fasting blood glucose < 125 mg/dL. Both habitual (≥ 1 cup/day) and nonhabitual (0 cup/day) coffee consumers were eligible. Exclusion criteria were evaluated using a health screening questionnaire (Qualtrics XM) which included (1) pre-existing conditions such as high cholesterol, hypertension, cardiovascular disease, gastrointestinal motility disorders (e.g., intestinal pseudo-obstruction, irritable bowel syndrome (IBS), fecal incontinence, constipation, hypo- or hyperthyroidism, anemia, renal disease, liver disease, hepatitis B or C, cholestasis or cirrhotic liver disease, nonalcoholic fatty liver disease, chronic pancreatitis, acute pancreatitis (current or within the last year), diabetes type 1 or 2, and insulin resistance), (2) pregnancy, breastfeeding, or polycystic ovarian syndrome (for females only), and (3) regular smokers and drinkers (one or more servings per day) were also excluded from the study.

**Figure 1:**
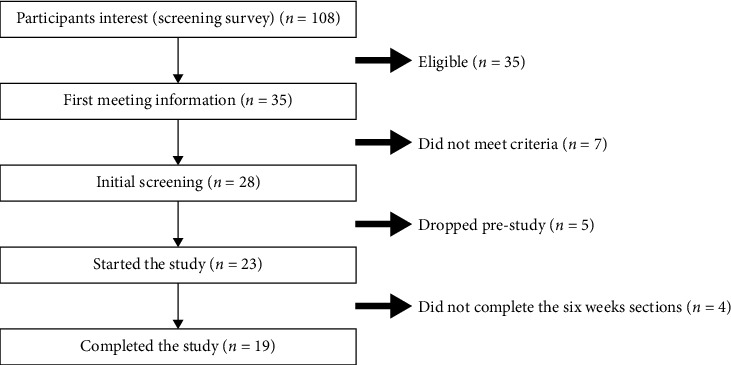
Study design flowchart.

Volunteers were recruited through flyers and e-mail invitations between January and February of 2020. In a preliminary session, subjects were informed of the purposes, experimental procedures, risks, and benefits of the study; then, a written informed consent was obtained from participants. A total of 108 participants were interested, and 30 participants consented to participate and were randomized to the treatments for the 6 weeks. A total of 29 subjects started the study, 19 subjects (15 female, 4 male) completed the study, and 10 subjects dropped the study for personal reasons through the six weeks treatment period (Figure 1). Subjects who completed the study were compensated financially at the study's conclusion.

### 2.2. Study Design

This study employed treatment combinations of coffee beverages at three roast levels (light, medium, and dark) and placebo given pre-, co- or postconsumption of 50 g of available CHO in the form of oral glucose tolerance test (OGTT) beverage or a standardized breakfast (B). This resulted in a total of three factors: coffee roast level, timing of coffee vs. CHO administration, and available CHO source.

Treatment combinations for this 6-week study ran in the following order: week 1: pretreatment + OGTT (PreO); week 2: pretreatment + breakfast (PreB); week 3: cotreatment + OGTT (CoO); week 4: cotreatment + breakfast (CoB); week 5: posttreatment + OGTT (PostO); and week 6: posttreatment + breakfast (PostB). Subjects participated once per week and were randomized each week to a coffee beverage or a noncoffee (placebo). Treatment combinations were replicated in triplicate each week.

### 2.3. Experimental Beverages and Food

Each treatment involved consumption of 300 mL of a test beverage, consisting of light, medium, or dark-roast coffee, or a noncoffee placebo (water with caramel color added). The OGTT beverage was a 150 mL glucose-sweetened fruit flavored punch (Thermo Scientific, USA). The standardized breakfast meal consisted of 42 g of corn flakes cereal (Kellogg's Corn Flakes Cereal, USA) and 300 mL of skim milk (Food Lion, USA). The choice of cereal flakes and skim milk reflected work by [12]. Both the OGTT and the standardized breakfast contained 50 g of available CHO, calculated from nutritional label values. OGTT and cereal with skim milk, which provided equivalent amounts of available glucose, were used in order to compare the effects of liquid versus solids foods on glucose responses when coffee was provided before with or after carbohydrates. The comparison of carbohydrate sources reflects the work of [26].

Coffee beverages were brewed from a single lot of green Arabica coffee beans roasted to light, medium, or dark levels at Counter Culture Coffee roasters (Durham, NC, USA). Coffee was ground using a VARIO-W (Baratsa, Italy), setting of 5E, and sieved (Sieve shaker Gilson, model ss-15, USA) to limit particle sizes to between 600 and 1000 μm. A drip brew paper-filtered method (MR Coffee, USA; Coffee maker, and Melitta, USA, Coffee Filters Natural Brown Unbleached) was used to produce coffee samples. Paper filters were used to limit extraction of the diterpenes, cafestol, and kahweol, into the coffee brewed for this study. These diterpenes have been reported to raise cholesterol levels when unfiltered coffee is consumed. Unfiltered Turkish coffee has been reported to contain 88 and 89 mg/L of cafestol and kahweol, respectively. In contrast, a second study reported 0.12 and 0.14 mg/L concentrations of cafestol and kahweol in filtered coffee [27, 28]. The coffees used in this study were brewed using a ratio of 55 g ground coffee per liter of deionized water, according to SCA standard methods for brewing coffee [29]. The placebo was a 1.6 g/L solution of caramel color (Durkee, USA) in water designed to mimic the color of brewed coffee. Each participant received 300 mL of test beverage.

### 2.4. Test Beverages Analysis

Color analysis was performed on whole and ground coffee as well as brewed coffee beverages using a Colorflex EZ Spectrophotometer colorimeter (HunterLab, model CFEZ 2636, USA). Color analyses used the LAB color model, where *L*^∗^ represented lightness (100 = white or 0 = black), *a*^∗^ represented green (negative values) versus red (positive values), and *b*^∗^ represented blue (negative values) versus yellow (positive values) [30]. Total percent dissolved solids (%TDS) and degrees Brix (°Brix) of brewed coffee beverages were measured using a digital refractometer (ATAGO PAL, Japan) [30]. Analyses of caffeine and CGA (as 3-, 4-, and 5-caffeoylquinic acid) were performed on brewed coffee samples following the method of [31] with minor modifications. Quantitative analysis was accomplished via a Waters Breeze high-pressure liquid chromatography (HPLC) system (Waters Corporation, USA) equipped with a Waters X-Bridge C-18, 3.5 μm column (100 × 4.6 mm), an autosampler, and a dual-channel UV/vis detector. Aliquots of 3 mL of brewed coffee were pipetted into conical centrifuge tubes, and then, 0.2 mL each of Carrez I and II aqueous solutions (potassium ferrocyanide, 3.6% w/v and zinc sulfate, 7.2% w/v, respectively) were added. Afterward, 4.6 mL of methanol were added, and samples were vortexed for 30 s. The mixtures were centrifuged at 500 rpm for 10 min. Sample supernatants were filtered through a 0.45 μm Tuffryn membrane Acrodisc syringe filter (Pall Corporation, Ann Arbor MI) into 1 mL vials and placed into an autosampler. Two mobile phases were used for HPLC separations. Mobile phase A was 90% 20 mM citric acid monohydrate in water and 10% methanol (v/v). Mobile phase B was 100% methanol. A linear gradient was used for separations, starting with 100% mobile phase A and transitioning to 100% mobile phase B over 25 min, at a flow rate of 0.6 mL/min. Caffeine and trigonelline were monitored at 276 nm, while CGAs (3- and 5-caffeoylquinic acids, abbreviated 3CQA and 5CQA) were monitored at 325 nm. Sample concentrations were quantified by peak area based on standard curves for caffeine, trigonelline as well as for 3CQA and 5CQA. Total phenol content was measured by the Folin–Ciocalteu method using gallic acid as standard, as described in [32]. The standards and chemical reagents were purchased from Sigma-Aldrich (USA). All test beverage analyses were conducted in triplicate.

### 2.5. Experimental Protocol

Tests were scheduled in the morning after an overnight (8–12 h) fast. Subjects were required to complete a short survey on each treatment day indicating that they had not engaged in activities that might interfere with blood glucose measures. Alcohol intake was restricted 24 h prior to each treatment, while other factors (nonprescription medications, cigarette smoking, and intense exercise) were restricted only during the overnight fast. Habitual caffeine use was not restricted except for the 8–12 h fasting time the night prior to each test. This was designed to prevent caffeine withdrawal symptoms that may have interfered with readings. Subjects were asked not to increase or decrease habitual caffeine intake over the course of the study.

**Figure 2:**
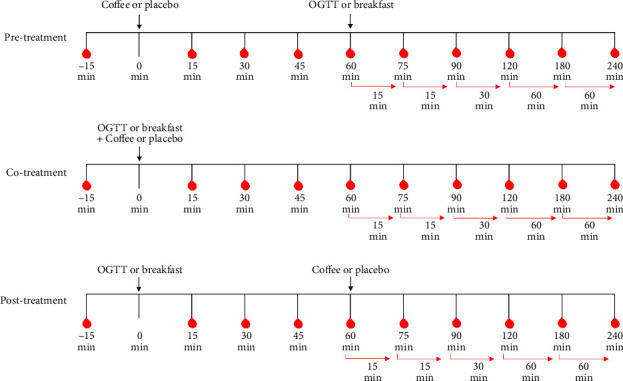
Overview of pre-, co-, and posttreatments, including timings of coffee and CHO intake, as well as blood glucose sampling times. Coffees, placebos treatments, OGTT beverages, and breakfasts were all consumed within 10 min of designated time points.

A capillary (finger-prick) fasting blood sample and the completion of a short survey were required of participants 15 min before the start of beverage consumption on each test day. Participants stayed in a quiet room to avoid physical exercises. Computers, mobile phone, and books were permitted.  Figure 2 shows the order of treatments and the timings of blood samples.

In the pretreatment + OGTT (PreO) or + Breakfast (PreB), participants were required to drink the test beverages (light, medium, and dark brewed coffee or placebo) at time zero (*t* = 0). Blood samples were taken 15, 30, 45, and 60 min after coffee consumption. Immediately after the 60 min blood sample, an OGTT beverage (150 mL) or a standardized breakfast meal was consumed; subsequent blood samples were taken at 75, 90, 120, 180, and 240 min (time from *t* = 0). In the cotreatment + OGTT (CoO) or + Breakfast (CoB), participants consumed the test beverage along with an OGTT (150 mL) or a breakfast meal at *t* = 0, and then, blood samples were taken at 15, 30, 45, 60, 75, 90, 120, 180, and 240 min. In the posttreatment + OGTT (PostO) or + Breakfast (PostB), participants consumed an OGTT (150 mL) or a breakfast meal at *t* = 0, and then, blood samples were taken at 15, 30, 45, and 60 min. After the 60 min blood sample was taken, participants consumed the test beverages (light, medium, or dark coffee levels, or placebo). Blood samples continued to be taken at 75, 90, 120, 180, and 240 min (time from *t* = 0). Across the study, all coffee, placebo, and OGTT beverages, as well as standard breakfasts, were consumed within 10 min. In the case of the CoO and CoB treatments, carbohydrates were consumed in first 5 min, and coffee beverages were consumed in the following 5 min to maintain a 10-min time window.

Blood glucose values were determined by finger-prick blood samples, using self-retracting safety lancets and calibrated glucometers (Nova Max Plus, USA). Subjects were trained to take their own blood glucose samples prior to the study. Blood glucose data were collected immediately after blood sampling using either paper or digital (Qualtrics XM) ballots.

### 2.6. Calculations and Statistical Analysis

Few studies have compared the effects of coffee beverage interventions on blood glucose responses following OGTT beverages versus standardized breakfasts. We, therefore, estimated sample size based on the work of [33] who reported blood glucose AUC values of 178 ± 52, 274 ± 45, and 201 ± 47 mmol/L ∗ 2 h for control, dark roast, and light roast coffee treatments, respectively. Based on this previously reported variation around AUC mean values, we utilized R software (v4.1.2; R Core Team 2021) to calculate that 12 subjects total would be required to detect a 20% difference in AUC values, with 80% power, at a Type I error level of 5%.

The study employed a placebo-controlled, single-blind, randomized design. One team member generated the random allocation sequence, and enrolled and assigned participants to treatments. All other team members, as well as participants, were blinded to treatment assignments. Subjects were randomized to treatments weekly using a William's Square design as reported by [34]. This involved randomization of subject numbers, then assigning subjects to treatments based on a William's Square randomization order. Treatments were combinations of three factors: coffee, timing, and food, as described previously. Treatment conditions were structured as a three-factor factorial design, comprising combinations of (1) coffee roast level (light, medium, or dark), (2) timing of coffee intake relative to CHO consumption (pre-, co-, or posttreatment), and (3) type of CHO source (OGTT or standardized breakfast). Participants completed randomly assigned treatment combinations over the course of the 6-week study, such that each three-factor combination was replicated a minimum of five times.

**Figure 3:**
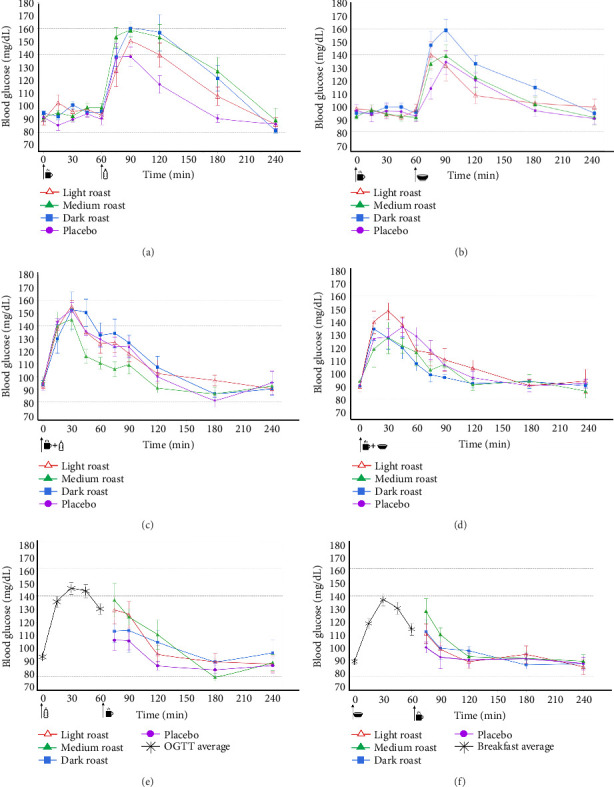
Blood glucose curves over 240 min showing CHO (bottle 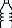 and bowl 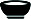 icons for OGTT and breakfast, respectively) and coffee timings (coffee cup 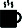 icon), across roast levels. PreO (a), pretreatment with coffee at *t* = 0 min, then OGTT beverage at 60 min. PreB (b), pretreatment with coffee at *t* = 0 min, then a standardized breakfast at 60 min. CoO (c), cotreatment with coffee and OGTT at *t* = 0 min. CoB (d), cotreatment with coffee and a standardized breakfast at *t* = 0. PostO (e), posttreatment with coffee at *t* = 60 min, after OGTT at *t* = 0 min. PostB (f), posttreatment with coffee at *t* = 60 min after a standardized breakfast at *t* = 0 min. Data are expressed as the mean ± standard deviation (*n* = 19).

**Figure 4:**
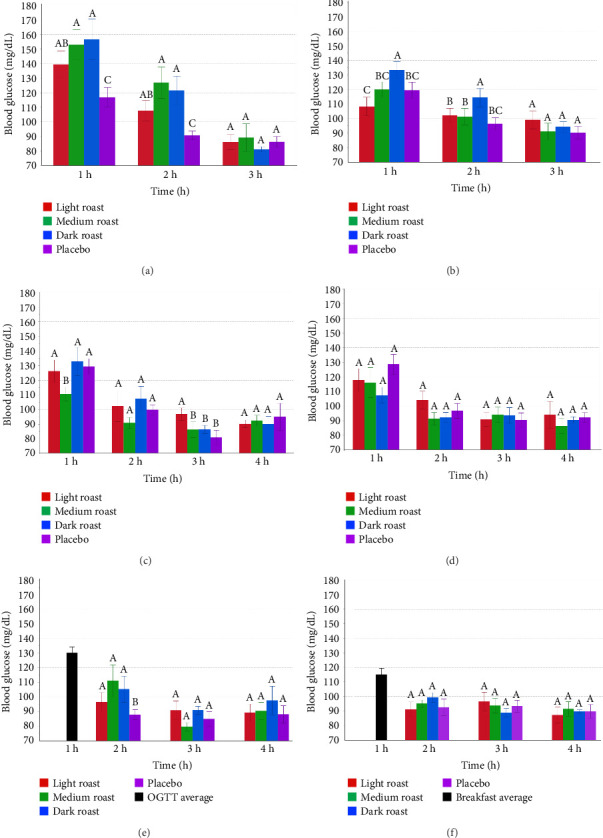
Postprandial peak blood glucose values (mg/dL) postcarbohydrate intake from 1 to 3 h for PreO (a), and PreB (b), and from 1 to 4 h for CoO (c), Co-Breakfast (d), PostO (e), and PostB (f) treatment conditions. Carbohydrates were consumed 1 h after coffee (a and b), at 0 min, along with coffee (c and d) or 1 h before coffee at time 0 (e and f).

Blood glucose values were measured at 10 time points out to 4 h across all treatment combinations (Figure 3) in order to determine peak blood glucose values and AUC. Postprandial peak blood glucose values were measured for either 3 h (PreO and PreB) or 4 h (CoO, CoB, PostO, and PostB) based on whether coffee was consumed prior to (Pre) along with (Co) or after (Post) OGTT or breakfast-based CHO sources (Figure 4). One of the 10 time points was used to determine whether participants met the Centers for Disease Control and Prevention's (CDC) definition of hyperglycemia, defined as blood glucose values > 140 mg/dL 2 h post-CHO ingestion [35, 36].

**Figure 5:**
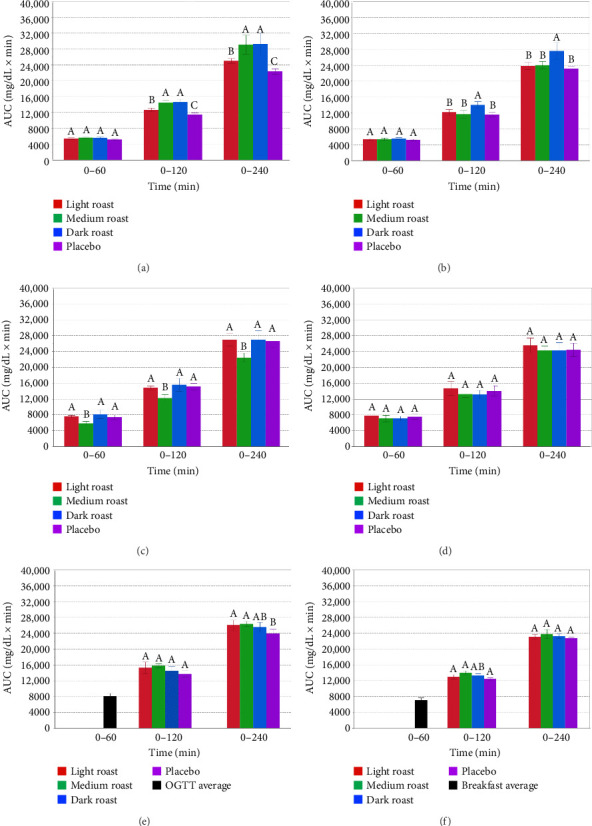
Blood glucose area under the curve (AUC) values for 0–60 min, 0–120 min, and 0–240 min time periods across all treatment combinations: PreO and PreB, (a, b), CoO and CoB (c, d), as well as PostO and PostB (e, f), in response to placebo, light, medium, and dark roast coffee beverages. Values are means ± SEM, *n* = 19. Data were analyzed using a one-way ANOVA for repeated measures with differences identified using the Tukey–Kramer post hoc analysis. Within each of the six treatment combinations and times (e.g., (a), 0–60 min), bars with the same letter above are not significantly (*p* < 0.05) different from one another.

Incremental AUC values for each treatment combinations (PreO, PreB, CoO, CoB, PostO, and PostB) were calculated using MATLAB version 9.13 (Mathworks Inc.) to integrate the curve by parts and sum the positive areas of each, based on the method of [37]. Areas under baseline (defined as mean blood glucose values at time 0) were not considered for curve by parts calculations. AUC (0–60, 0–120, and 0–240 min) results are shown as postprandial plasma glucose responses to OGTT and breakfast and are reported in Figure 5. Tukey adjustments were used for post hoc pairwise comparisons according to [11]. A multivariate analysis of variance (ANOVA) was used to compare peak and AUC glucose responses among the three factors, as test beverages light, medium, and dark roast coffee and placebo, type of treatment pre-, co-, and post, and type of food OGTT and breakfast. The data for this study were determined to be normally distributed, based on the Shapiro–Wilk test for normality (*p*=0.73). Significant differences among means were determined using Tukey's test to adjust for multiple comparisons. Significance was set at *p* < 0.05. R statistical software (v4.1.2; R Core Team 2021) was used for all statistical analyses.

## 3. Results

### 3.1. Coffee Beverage Analyses

Participants consumed 300 mL of coffee during each visit, representing 217.80 ± 6.6, 220.50 ± 8.1, and 238.20 ± 6.9 mg of caffeine for light, medium, and dark roast, respectively. Conversely, 3CQA (the primary CGA in our coffee beverages) levels were 278.40 ± 5.1, 117.60 ± 6.6, and 29.70 ± 2.1 mg for light, medium, and dark roast, respectively, for a 300 mL beverage. The placebo contained neither caffeine nor CGAs.

**Figure 6:**
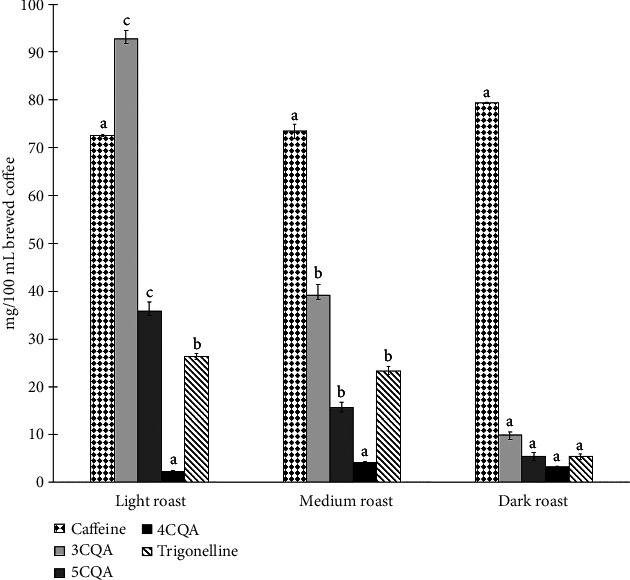
Comparison of caffeine, 3CQA, 4CQA, 5CQA, and trigonelline concentrations by roast level. Values shown are mg/100 mL brewed coffee. Values are means ± standard deviations. Bars for a given analyte labeled with the same lower-case letter do not differ significantly (*p* > 0.05).

As shown in Figure 6, caffeine concentrations ranged from 72.60 ± 2.2 to 79.40 ± 2.3 mg/100 mL in brewed coffees but did not differ significantly across light, medium, and dark roast levels. In contrast, concentrations of 3CQA, 5CQA, and trigonelline were significantly higher for light roast brewed coffees (92.80 ± 1.70, 35.9 ± 1.90, and 26.28 ± 0.16 mg/100 mL), than for medium (39.30 ± 2.20, 15.60 ± 1.2, 23.33 ± 1.49 mg/100 mL) or dark roast (9.90 ± 0.70, 5.50 ± 0.70, 5.38 ± 0.18 mg/100 mL). Concentrations of 4CQA were low but stable across light, medium, and dark roast brewed coffees (2.30 ± 0.20, 4.10 ± 0.30, and 3.20 ± 0.20 mg/100 mL, respectively) with no significant (*p* > 0.05) differences based on roast.

Table 1 describes the chemical and physical properties of the coffees used in this study beyond caffeine, CGAs, and trigonelline.

As seen in Table 1, *L*^∗^, *a*^∗^, and *b*^∗^ color values, as well as total phenolic values, showed a clear decreasing trend from light to medium to dark roast, wherein each value decreased significantly (*p* > 0.05) from one roast to the next. Only °Brix and TDS (%) values did not follow this trend, with the highest values observed with dark roast brewed and lowest values observed with medium roast brewed coffees.

### 3.2. Subject Characteristics

There were 108 responses to the initial screening survey. Of the 108 respondents, a total of 30 subjects were recruited into the study. Of the 30 recruited, 19 participants (15 females and 4 males) completed the study. The baseline characteristics of those who completed the study are summarized in Table 2. The average age and BMI of female participants were 25.6 ± 0.2 years and 23.67 ± 0.31 kg/m^2^, respectively, while the average age and BMI for male participants were 27.3 ± 1.15 years and 23.98 ± 1.84 kg/m^2^, respectively. None of the participants reported physical discomfort or negative health outcomes or any concerns related to compliance with the study protocol. Based on surveys completed before each test day, no activities that may have interfered with blood glucose data values were reported by study participants over the 6 weeks of the study.

Both habitual coffee consumers *n* = 13 (1–4 cups/day) and noncoffee consumers *n* = 6 (≤ 1 cups/day) participated in this study. All subjects met the study fasting blood glucose requirement ≤ 100 mg/dL.

### 3.3. Study Baseline

Average fasting blood glucose responses at *t* = 0 min across all treatments were not significantly different (93.5 ± 1.56 mg/dL) (*p* > 0.05) from each other, providing a stable baseline from which to estimate treatment effects.

### 3.4. Blood Glucose

Figure 3 provides an overview of blood glucose responses over 240 min for all treatment timings, where coffee was given prior to (Pre-) along with (Co-) or after (Post-) an OGTT beverage or a standardized breakfast. The subsections that follow provide greater detail about results for specific time versus CHO source combinations.

#### 3.4.1. Pretreatment-OGTT and Standardized Breakfast (PreO and PreB)

Placebo consistently results in lower blood glucose peaks than the coffee beverages across the treatments. Coffee beverages regardless of roast level did not significantly (*p* > 0.05) affect blood glucose responses in the PreO and PreB treatment conditions during the 60 min before consuming a glucose source (Figures 3(a) and 3(b)). However, following the administration of OGTT or standardized breakfast, blood glucose concentrations increased rapidly, for all beverages including the placebo.

Despite higher mean values for dark, medium, and light roast coffee, relative to the placebo (159.7 ± 5.7 mg/dL, 158.3 ± 12.5, and 150.0 ± 10.6 vs. 138.14 ± 7.7 mg/dL), respectively, at *t* = 90 min, no significant differences were observed across treatments (*p* > 0.05) in the PreO conditions peak glucose levels. In contrast for PreB treatments, dark roast coffee alone showed significantly higher mean values (158.8 ± 8.4 mg/dL vs. 134.0 ± 9.2 mg/dL) relative to placebo at *t* = 90 min (*p* < 0.05) (Figure 3). After the 90 min peak, blood glucose concentrations steadily declined and returned to baseline at *t* = 240 min for both treatments PreO and PreB conditions.

#### 3.4.2. Cotreatment-OGTT and Standardized Breakfast (CoO and CoB)

In the CoO and CoB conditions where coffee was consumed with an OGTT beverage or a standardized breakfast at *t* = 0 min, no significant differences were observed for coffee treatments and placebo in the CoO treatment. However, in the CoB condition, light roast coffee alone showed significantly higher mean values of 151.8 ± 9.7 mg/dL versus 127.8 ± 6.9 mg/dL of placebo (*p* < 0.05) at *t* = 30 min. All treatments returned to baseline at *t* = 180 min in the CoO and CoB treatment groups (Figure 3).

#### 3.4.3. Posttreatment-OGTT and Standardized Breakfast (PostO and PostB)

In the PostO and PostB conditions, the OGTT or the standardized breakfast was consumed by participants 60 min before coffee beverages. Like CoO and CoB treatments, glucose values peaked at *t* = 30 min. No significant differences were observed across treatments at any time points PostO and PostB, despite higher values obtained for medium roast coffee in 13.34% and 12.49% (124.3 ± 5.9 and 111.4 ± 4.8) mg/dL versus placebo (106.2 ± 6.6 and 94.5 ± 8.5) mg/dL, respectively (Figure 3).

### 3.5. Peak Blood Glucose Values

Figure 4 shows the average peak blood glucose concentrations calculated for each time point in response to CHO (OGTT or breakfast) intake. All OGTT conditions are shown on the left (Figures 4(a), 4(c), 4(e)). Breakfast conditions are shown on the right (Figures 4(b), 4(d), 4(f)). Figure 4 shows postprandial peak blood glucose values at one, two, three, and 4 h for Pre-, Co-, and Post-coffee timing conditions across light, medium, and dark roast coffees, where OGTT or breakfasts served as CHO sources. Overall, the highest peak blood glucose values were observed in the PreO treatment combination 1 h after CHO consumption across all coffee roast levels (Figure 4(a)). Peak blood glucose values were generally higher when OGTT was used as a CHO source (Figures 4(a), 4(c), 4(e)) than glucose values after a standardized breakfast (Figures 4(b), 4(d), 4(f)).

The effects of roast level were dependent on the timing of coffee versus CHO consumption. Across pretreatments (Figures 4(a) and 4(b)), roast had no significant effect, with the exception of significantly higher blood glucose values with dark roast in the PreB condition at the 2 h time point. For CoO conditions, medium roast coffee showed significantly low peak blood glucose values at 1 h, while light roast gave significantly higher values compared to other treatments at the 3 h time point. No significant differences were observed among coffee and placebo treatments at any time point for the Co-Breakfast condition. In the PostO condition, peak blood glucose values were significantly lower for the placebo at 2 h only. In the PostB condition, no significant differences were observed among coffee and placebo treatments at any time point. Two hours after either OGTT or breakfast consumption, all blood glucose peaks fell below 130 mg/dL, across all treatment combinations, while after 3 h, all peak blood glucose values fell below 100 mg/dL.

### 3.6. Incremental AUC

Figure 5 shows a comparison of AUC (0–60 min, 0–120 min, and 0–240 min) values of all treatment combinations across three time points for PreO and PreB (Figures 5(a) and 5(b)), CoO and CoB (Figures 5(c) and 5(d)), as well as PostO and PostB (Figures 5(e) and 5(f)). For PreO and PreB combinations, where coffee was provided prior to CHO sources, AUC (0–60 min) values were lower than for either Co- or Post-treatment timings, where coffee was provided with or after CHO, respectively. In the PreO combination (Figure 5(a)), light roast coffee showed significantly (*p* < 0.05) lower values at AUC (0–120 min and 0–240 min) than medium or dark roast coffee, but not as low as the placebo at either time point. For the PreB combination (Figure 5(b)), AUC values for light and medium roast coffees were the same as for the placebo and dark roast showed the highest values overall for AUC (0–120 min and 0–240 min). In the CoO combination (Figure 5(c)), medium roast coffee showed significantly (*p* < 0.05) lower AUC values compared with other treatments across all three time points. No differences were observed among light, dark, or placebo treatments in the CoO combination. For the CoB combination (Figure 5(d)), no differences were observed across treatments for all three time frames. For the PostO and PostB combinations (Figures 5(e) and 5(f)), no differences were observed across coffee and placebo treatments for the AUC (0–120 and 0–240 min) time frames. Note that the single bar for PostO and PostB AUC (0–60 min) time frames is due to the administration of OGTT beverages or standardized breakfasts, respectively, 60 min prior to coffee or placebo intake. Overall, with the exception of medium roast coffee in the CoO combination, coffee taken with or after CHO did not affect blood glucose levels.

Table 3 shows main effects and interactions for AUC data. All three main effects (CHO type, coffee roast, and coffee timing) were highly significant (*p* < 0.002). Two AUC interactions (coffee timing × coffee roast and coffee timing × CHO type × coffee roast) were highly significant (*p* < 0.0001), while two other interactions (coffee timing × CHO type and CHO type × roast level) were not significant (*p* > 0.05). Table 3 emphasized that CHO type (especially OGTT), coffee roast level (especially medium and dark), and the timing (especially pre-timings) of coffee intake relative to CHO were all important and that combinations of these factors also drove blood glucose AUC values. These interactions were especially evident in the PreO and PreB combinations shown in Figure 3, where medium and dark roast coffees produced higher AUC values than light roast coffees.

## 4. Discussion

This human clinical trial examined the effects of coffee roast level, the timing of coffee consumption, and available CHO source on blood glucose values over 240 min. Glucose values were analyzed in terms of blood glucose curves (Figure 3), peak postprandial blood glucose (Figure 4), and incremental AUC (0–60, 0–120, and 0–240 min) (Figure 5). There is a growing body of literature on clinical trials examining the health effects of coffee, but few studies have examined the effects of the physical and chemical changes brought about by roasting on blood glucose responses in healthy humans.

Coffee is a complex matrix, containing numerous bioactive compounds that vary with roasting. We observed that caffeine and 4CQA were stable to roasting, but that trigonelline, 3CQA, 5CQA, and total phenol concentrations decreased. Pronounced shifts in *L*^∗^, *a*^∗^, and *b*^∗^ color values also occurred with increasing roast level (Figure 6, Table 1). TDS and °Brix, brew strength metrics which relate to sensory properties like richness and body, were significantly higher in dark roast coffee, likely due to greater extractability of solids during brewing [29, 38–40]. Other authors have reported similar chemical and physical changes with roasting [21, 33, 39, 41, 42].

Blood glucose curves, postprandial peak values, and AUC provided distinct, but complimentary information about coffee treatments. Blood glucose curves demonstrated that postprandial glycemia increased rapidly after CHO consumption across all treatment combinations (Figure 3). PreO and PreB treatments (Figures 4(a) and 4(b)) produced peak glucose values as high as 160 mg/dL 30 min after CHO intake, a pattern not observed in Co- or Posttreatment conditions where all other values fell below 155 mg/dL at that time point. These values were higher than the 8 mmol/L (144 mg/dL) values reported by [33] at 30 min post-CHO intake. While blood glucose peaks reaching 160 mg/dL were observed at 30 min post-CHO ingestion in the PreO and PreB combinations, at 2 h post-CHO intake, all participant values fell below 130 mg/dL in the Pre-treatments and below 110 mg/dL in the Co- and Post-treatments.

Our study participants were normoglycemic based on fasting glucose levels (< 100 mg/dL), BMI, and other health indicators, but the observed transient spikes above 140 mg/dL could raise questions about their potential physiological significance, in light of growing evidence about the importance of postprandial peak glucose values, rather than fasting glucose levels alone. The authors of [43] reported that post challenge glucose spikes were more strongly associated with cardiovascular risk than fasting glucose levels. Their data indicated that the 2-h OGTT glucose level was the strongest independent predictor of cardiovascular risk in individuals with diabetes. The elevated glucose responses observed at 30 min in the PreO and PreB combinations in our study may have represented early metabolic dysregulation in some individuals or may have reflected individual variation in response to combined caffeine and CHO ingestion as well as the timing of glucose measurements. While the CDC defines hyperglycemia in prediabetic conditions as blood glucose levels exceeding 140 mg/dL 2 h after a glucose challenge, emerging research emphasizes the importance of assessing postprandial peak “glucotypes” in clinical trials. Glucotypes refer to interindividual variability in glycemic responses [36].

Coffee consumed prior to CHO in PreO and PreB treatments in a fasted state did not elevate blood glucose alone but did potentiate higher glucose peaks after CHO intake, at caffeine doses as low as 100 mg. These findings are consistent with several previous studies [8, 12–14, 44]. They are in partial agreement with others who also analyzed both CGA and caffeine content for light, medium, and dark roast coffee and reported that dark roast coffee increased peak glucose without significantly affecting AUC relative to a water control when OGTT was provided as a CHO source after coffee [33]. The observed higher blood glucose levels in PreO and PreB treatments could be attributed to elevated catecholamine levels, impaired peripheral glucose uptake, and increased hepatic glucose production caused by caffeine [16, 44, 45]. Caffeine, an adenosine receptor antagonist, inhibits adenosine-mediated insulin signaling and glucose uptake, reducing peripheral glucose disposal and hepatic glucose suppression [16, 44, 46]. Reference [46] examined the effect of caffeine intake on glucose disposal in sedentary humans, finding that caffeine reduces insulin sensitivity and glucose disposal in resting individuals. Participants received either a dose of caffeine (5 mg/kg of body weight) or a placebo. In our study, the average caffeine dose was lower (3.4 mg/kg) than the amount used by the previous authors, but may still have impaired glucose regulation in the PreO and PreB conditions. Unlike the study by [46], we provided brewed coffee rather than pure caffeine to our participants, such that many other noncaffeine compounds were present in our test beverages.

Similar to peak glucose findings, coffee alone in a fasted state did not affect AUC values in PreO and PreB treatments. AUC values only increased after CHO was provided. In the PreO condition, where OGTT beverages served as CHO sources, light roast coffee showed significantly (*p* < 0.05) lower values than the placebo at AUC (0–120 and 0–240 min). Both medium and dark roast coffees, in contrast, significantly (*p* < 0.05) increased mean AUC values in the PreO condition. Only dark roast coffee significantly increased AUC values in the PreO condition, in which a standardized breakfast was provided. There is no universally agreed upon definition for light versus medium versus dark roast coffee, but our observations suggest that darker roasted coffees consumed prior to CHO potentiated increases in blood glucose AUC and that light roast coffees inhibited increases in blood glucose AUC when OGTT. Potentiating effects of darker roasted coffees when consumed prior to CHO may relate to caffeine-promoted insulin resistance unopposed by acarbose-like molecules [47–52]. Arguments in favor of the inhibitory effects of light roast coffee on blood glucose AUC in the PreO condition could relate to the higher concentration of acarbose-like molecules, such as caffeic acid and CGA, as well as trigonelline [53]. Some studies have reported increased postprandial glucose AUCs for coffee versus placebo [12, 54] while others have not [6, 46, 55]. A study providing CGA and trigonelline in decaffeinated coffee to overweight participants showed blood glucose concentrations were significantly lower compared with placebo for only 15 min after an OGTT but did not significantly reduce the AUC [19]. The authors also found that decaffeinated coffee did not significantly change mean blood glucose concentrations at any time point over the 120 min of measurement, again raising the possibility that caffeine may have driven the elevated glucose level observed in PreO and PreB combinations in our study, and this also was observed by [6].

When coffee was co-consumed with CHO, fewer differences in glucose responses were apparent, relative to pretreatments. In terms of glucose curves and peaks, cotreatment resulted in lower glucose levels overall at 30 min (Figure 3) and at 60 min (Figures 3 and 4). Interestingly, light roast peak glucose values were significantly (*p* < 0.05) higher than all other treatments at the 180 min in the PreO condition. Looking at Figure 3 glucose curves, this seems to have resulted from a more gradual decrease in blood glucose levels over time. In terms of AUC, differences were noted only in the CoO condition, medium roast (Figure 5(c)). AUC values for medium roast coffee were significantly (*p* < 0.05) lower than other treatments at all three AUC (0–60, 0–120, and 0–240 min) time frames measured in the CoO combination alone (Figure 2). One potential explanation for this observed effect on AUC is that medium roast may represent a middle ground where acarbose-like molecules present in lighter roasts are partly preserved, while the soluble fiber that is more accessible in darker roasts may also have had an effect [56]. Despite a higher concentration of acarbose-like molecules, such as CGA and trigonelline, which other authors have reported to improve glucose regulation, light roast coffee did not perform differently than dark roast or placebo across AUC glucose measurements [19].

Since no differences were observed at any time point across all treatments in the CoB combination, this indicates that the effects of co-consumption of coffee on AUC differed when CHO sources were provided as glucose in liquid form (OGTT) versus when provided as a semisolid food (standardized breakfast with liquid nonfat milk and solid corn flakes), even when those CHO sources were matched for available glucose. An observed difference in the effects of liquid versus solid or semisolid foods on blood glucose responses would be in agreement with other reports in the literature, although results are not consistent in terms of glycemic effects of liquid versus solid foods [57, 58]. Our results were partially aligned with the work of [55], who reported that coffee had no effects on glycemic responses when co-consumed with CHO, although they did report lower glycemic responses for both a starch-containing solid food (breakfast bun), as well as sucrose and milk, versus pure glucose. This suggests that the effects of coffee chemistry on blood glucose responses may vary, depending on the timing of coffee consumption, relative to CHO, and the type of CHO in question, in fasted versus nonfasted states.

For Posttreatments, in which coffee was consumed 60 min after CHO, blood glucose curves and peaks (Figures 3(d) and 4(d)) showed higher glucose values for OGTT than for the standard breakfast, indicating a differential effect of liquid versus solid CHO sources, as mentioned previously. Coffee had no significant effects on blood glucose AUC in Post-timings (PostO and PostB, Figures 5(e), 5(f)) regardless of roast level. The observed effects in the PostO and PostB combinations may have been due to the high bioavailability of the CHO sources in the OGTT and the standardized breakfasts, such that the natural processes of glucose absorption were already well underway when the coffee was consumed. Figures 3 and 4 support this explanation, since they show that the highest blood glucose values were achieved within 1 h of CHO ingestion. The most general finding, relative to the effects of coffee on AUC, was that the consumption of coffee in nonfasted states (along with or after CHO) has minimal effects on blood glucose, regardless of coffee roast level.

Coffee's chemistry is paradoxical in relation to its effects on blood glucose, based on its content of caffeine, acarbose-like molecules, and soluble fiber. While caffeine is known to reduce insulin sensitivity and potentially raise blood glucose, this effect may not be constant across all roast degrees. Light roast coffees may demonstrate more acarbose-like activities, based on higher CGA content, relative to medium and dark roasts, as evidenced by the effects of light roast coffee on AUC data in the PreO condition. CGA has been reported to inhibit intestinal glucose absorption [9]. We hypothesize that an inhibition of glucose absorption could explain why light roast coffee showed higher peak glucose at 180 min post OGTT in the CoO condition, relative to all other treatments without increasing AUC at any time point over 240 min, suggesting a lower glycemic impact overall.

Coffee beverages are a source of soluble dietary fiber, primarily in the form of galactomannans and type II arabinogalactans [56]. According to [59], espresso preparations contained between 375 and 654 mg/100 mL of soluble fiber, while drip-brewed coffee prepared using paper filters (as in the present study) contained slightly lower amounts, ranging from 259 to 369 mg/100 mL. The authors found that dark-roast coffees exhibited higher levels of extractable soluble fiber compared to light roasts, and this is potentially due to the formation of more fragile, glass-like cell wall structures during prolonged roasting [60]. Although soluble fiber content was not directly measured in our study, its potential contribution to the observed effects of coffee on postprandial blood glucose responses warrants consideration. Soluble fibers such as galactomannans and arabinogalactans have been shown to inhibit CHO-digesting enzymes like α-amylase and α-glucosidase, which could attenuate glucose absorption [48, 61]. We observed both physical and chemical differences across roast levels in this study including color, TDS, trigonelline, CGA, and total phenolic content support the idea that both chemical and physical changes may contribute to a coffee's glycemic effects.

We measured higher TDS (dissolved solids) in dark roast coffee than in lighter roasts. Dark roast coffee also produced higher blood glucose levels in our study. Since other authors have reported associations between dark roast coffee and elevated blood glucose in humans, we propose that future clinical studies identify the types of dissolved solids in coffee and their biological effects. A free-living study of 118 overweight participants [62], compared the effects of consuming four to five cups of dark roast, which the authors characterized as high in N-methylpyridinium with medium roast, characterized as high in CGAs and trigonelline (the precursor to N-methylpyridinium) over a period of three months. While no acute effects on blood glucose were reported for either roast level, the authors observed an increase in HbA1c (glycated hemoglobin) a marker of long-term glucose regulation following consumption of the dark roast. In our study, we previously reported a decrease in trigonelline, 3-CQA, 5-CQA, and total phenolic content with increasing roast level. These findings raise the question of whether the observed effects on blood glucose were due to an increase in soluble compounds such as N-methylpyridinium or to a loss of acarbose-like and other types of bioactive compounds that may help control blood glucose.

This study had several key limitations that could drive other future studies. First, the sample size was relatively small, with 29 participants starting and 19 finishing the study. Despite the attrition of subjects, treatment combination was replicated in triplicate across the study, allowing for statistically significant differences to be detected among treatment combinations. Second, the participants were comprised of 15 women and 4 men. Given reports of sex differences in AUC glucose responses when caffeine is coadministered with CHO [8], any follow-up studies should have a closer parity of women and men to examine the interactions of CHO, caffeine, and sex on blood glucose responses. For future studies, it will be important to recruit a larger number of participants with a closer balance between the sexes. It may also be helpful to include a wider range BMIs, with the expectation that a wider range of “glucotypes” will be observed. Notably, in prior studies, normal-weight men did not show the same glycemic effects as overweight individuals, suggesting that biological differences may influence in the response to caffeine and CHO co-ingestion. Third, both caffeine/coffee consumers (*n* = 13) and nonconsumers (*n* = 6) completed the study. For caffeine consumers, we did not attempt to restrict customary caffeine intakes. Given that few differences in peak or AUC-based glucose measures were observed for consumers versus nonconsumers of caffeine/coffee, we did not view caffeine intake prior to our study as a major limitation. The inclusion of caffeine-only, CGA-only, and trigonelline-only controls at appropriate concentrations could further define the roles that these compounds play in glucose responses in future studies.

## 5. Conclusions

Our findings highlighted specific combinations of coffee roast level, timing of coffee consumption, and the type of available CHO that influenced blood glucose levels. To our knowledge, this is the first study to examine the effects of varying coffee roast degrees on peak and AUC glucose levels when consumed before, with, or after two different CHO sources. Light roast coffee given prior to CHO inhibited increases in blood glucose AUC when OGTT was the CHO source. In contrast, darker roast coffees consumed before CHO (both PreO and PreB) were most effective at raising both peak and AUC blood glucose values. Our data also indicate that coffee given with or after CHO did not affect blood glucose to the same extent as coffee given prior to CHO, emphasizing the importance of timing. Medium and dark roast coffees given prior to CHO potentiated elevated glucose levels. These interactions may have implications for individuals who may benefit from temporary elevations in blood glucose levels (for exercise performance and recovery) versus those seeking to moderate glucose levels. Consuming light roast coffee before CHO beverages as well as consuming coffee at any roast level with or after CHO may be helpful for those seeking to moderate blood glucose levels. This study emphasizes the complex interactions of multiple factors, such as CHO type, coffee roast level, and coffee timing relative to CHO intake on blood glucose responses in humans.

## Figures and Tables

**Table 1:** Physical and chemical characteristics of roasted coffee beans, ground coffees, and brewed coffees^1^.

Coffee sample	*L* ^∗^	*a* ^∗^	*b* ^∗^	Soluble solids (°brix)	%TDS	Total phenolic content (mEq GAE/100 g coffee)
Beans						
Light roast	27.60 ± 0.19a	7.49 ± 0.23a	13.43 ± 0.38a	—	—	—
Medium roast	22.24 ± 0.61b	6.88 ± 0.13b	9.35 ± 0.26b	—	—	—
Dark roast	17.68 ± 0.32c	4.68 ± 0.08c	4.60 ± 0.08c	—	—	—
Ground coffee						
Light roast	29.17 ± 0.35a	13.93 ± 0.10a	22.58 ± 0.29a	—	—	—
Medium roast	20.33 ± 0.25b	10.69 ± 0.01b	12.34 ± 0.06b	—	—	—
Dark roast	15.44 ± 0.21c	6.53 ± 0.06c	5.62 ± 0.09c	—	—	—
Brewed coffee						
Light roast	12.53 ± 0.21a	22.88 ± 0.18a	19.68 ± 0.40a	1.61 ± 0.04b	1.29 ± 0.04b	18.5 ± 0.85a
Medium roast	4.63 ± 0.13b	11.43 ± 0.41b	6.50 ± 0.09b	1.42 ± 0.15c	1.12 ± 0.12c	16.8 ± 1.09ab
Dark roast	2.56 ± 0.09c	3.43 ± 0.13c	2.82 ± 0.09c	1.68 ± 0.05a	1.33 ± 0.04a	15.5 ± 0.98b

*Note:* Data were analyzed using a one-way ANOVA for repeated measures with differences identified using the Tukey–Kramer post hoc analysis. Column values showing same the lower-case letter for the same type of coffee (beans, ground, and brewed) do not differ significantly *p* > 0.05.

^1^Values are means ± standard deviations.

**Table 2:** Baseline subject characteristics (*n* = 19)^1^.

Parameters	Value^1^
Age (y)	28.9 ± 2.62
Weight (kg)	65.86 ± 1.48
Height (cm)	166.88 ± 1.17
Body mass index (kg/m^2^)	23.63 ± 0.42
Waist circumference (cm)	79.84 ± 2.09
Hip circumference (cm)	98.50 ± 2.25
Waist-hip ratio	0.81 ± 0.01
Fasting blood glucose (mg/dL)	93.5 ± 1.56

^1^Values are means ± standard errors of the mean (SEM); *n* = 19.

**Table 3:** AUC comparisons of main effects and interactions among study factors by ANOVA (*p* < 0.05), for blood glucose response over 240 min.

Factors	*F*-value	*p -*value
Main effects		
Carbohydrate (OGTT, standardized breakfast)	39.61	< 0.0001^∗^
Roast (light, medium, and dark coffee)	11.72	< 0.0001^∗^
Timing (pre-, co-, or postcoffee)	6.82	< 0.002^∗^
Interactions		
Timing × roast	13.47	< 0.0001^∗^
Timing × carbohydrate × roast	5.59	< 0.0001^∗^
Timing × carbohydrate	1.34	0.266
Carbohydrate × roast level	1.25	0.294

^∗^Indicates that the *F*-values for main effects or interactions were statistically significant.

## Data Availability

This trial was registered at the NIH Clinical Trials Registry (clinicaltrials.gov) under the name, “Effects of Coffee Roasting on Blood Sugar Levels in Healthy Humans.” All data were reported in the study. All other individual participants' data are available upon reasonable request from the corresponding author.
